# The More Than Words® parent-delivered program for autistic children and those with social communication challenges: a single-arm pragmatic feasibility trial

**DOI:** 10.1186/s40814-026-01783-0

**Published:** 2026-02-20

**Authors:** Amanda V. Binns, Patricia Tucker, Lauren Denusik, Vipula Rajesh Kumar, Janis Oram

**Affiliations:** 1 School of Communication Sciences and Disorders, Western University, Elborn Building, London, ON N6A 1H1 Canada; 2 Department of Speech-Language Pathology, University of Toronto, Toronto, Canada; 3 School of Occupational Therapy, Western University, London, Canada; 4 Graduate Program in Health and Rehabilitation Sciences, Western University, London, Canada

**Keywords:** Autism, Social communication, Intervention, Parent coaching

## Abstract

**Background:**

Pediatric speech-language pathologists (SLPs) frequently use parent coaching interventions such as the *More Than Words*® (MTW) program to support young autistic children and others with social communication challenges. While some promising evidence exists for MTW, outcomes from previous studies have been mixed and research to date has primarily been conducted in controlled settings, limiting generalizability to real-world practice. To support future pragmatic randomized controlled trials (RCTs), this study aimed to assess the feasibility of a MTW evaluation protocol that had been co-constructed by researchers, program developers, and expert SLPs for use in community settings.

**Methods:**

This pragmatic, single-arm, pre-post feasibility study implemented the co-constructed evaluation protocol during MTW programs delivered by community-based SLPs as a part of standard care in a publicly funded service. We assessed the following dimensions of methodological feasibility: (a) recruitment capability; (b) feasibility, acceptability, and practicality of data collection and analysis procedures; and (c) preliminary outcome trends.

**Results:**

Of those approached, 45% of SLPs and 57% of parent–child dyads agreed to participate. Four of the five enrolled SLPs delivered their MTW program as planned and none of the 21 enrolled parent–child dyads withdrew from the study. Outcome measure completion ranged from 48 to 81%, with 4 dyads providing little to no data. SLPs viewed study methods and measures as acceptable and feasible and researcher coding of video-recorded outcome measures was reliable and practical. Preliminary outcome trend analyses indicated post-program gains in children’s early communication skills and communicative participation by parent report, and in child, parent, and dyadic interaction behaviors coded by the researchers. All parents agreed or strongly agreed that their child benefited from the MTW program and most reported increased self-efficacy in supporting their child’s communication.

**Conclusions:**

This feasibility trial offers a valuable contribution to advancing pragmatic clinical trial methods for use with MTW and other similar programs, highlighting both the challenges and benefits of moving beyond controlled experimental settings. Although findings may not fully generalize, new insights into participant recruitment, data collection, and methodological feasibility are applicable more broadly and can inform future pragmatic RCT development for evaluating effectiveness of community-based caregiver-delivered programs.

**Trial registration:**

The trial was retrospectively registered at ClinicalTrials.gov (ID: NCT06847347).

## Key messages regarding feasibility

- What uncertainties existed regarding the feasibility? We had uncertainties about study recruitment and retention, outcome measure completion rates, acceptability and feasibility of the measures and procedures for use during routine delivery of the program in community settings, and preliminary outcome trends of the program as evaluated using the selected outcome measures.
- What are the key feasibility findings? Roughly half of those approached agreed to participate and only one clinician dropped out. Most parent-child dyads had at least one complete pre-post outcome measure, although 20% had little to no data and individual measure completion varied between 48–81%. Speech-language pathologists viewed the measures and methods as acceptable and practical, and researcher coding of video-recorded outcome measures was reliable and feasible. Children and parents showed gains post-program on outcome measures, and parents perceived the program to be effective.
- What are the implications of the feasibility findings for the design of the main study? Our results indicate that recruitment for the main study is feasible, but we might anticipate at minimum 20% missing data. Findings suggested that the selected outcome measures captured meaningful outcome trends, were sensitive to change over time, and would be feasible and reliable for use in the main study. Overall, our findings suggest that it would be prudent to proceed next to a pilot rather than definitive RCT to assess potential factors affecting recruitment of the control group and randomization, as well as assess the impact of suggestions for increasing data completion rates generated during the current feasibility trial.

## Background

Pediatric speech-language pathologists (SLPs) report that a substantial proportion of their caseloads now include children with diagnosed or suspected autism [1]. This aligns with persistent increases in autism diagnoses worldwide [2] and underscores the importance of effective, feasible, and resource-efficient support services. For children with diagnosed autism, or children with social communication challenges who may be waiting for diagnostic assessments, identifying effective services targeting language and social communication is particularly important because development of these skills is among the most important contributors to long-term outcomes in autistic children [3]. Furthermore, supporting social communication aligns with parents’ treatment priorities for their autistic children [4–6].

SLPs provide early intervention services that focus on supporting children to engage, interact, and communicate with the people around them in meaningful contexts. For young children, their parents are often their primary communication partners. Therefore, parent coaching interventions are commonly used by SLPs to support social communication development in young autistic children [7]. Parent coaching interventions allow clinicians to capitalize on parents’ expert knowledge of their child. Additionally, these interventions increase the potential for children to generalize skills learned in sessions to their daily life, thus increasing treatment dosage without increasing the cost [8]. Furthermore, by reducing the frequency of direct contact hours with the therapist, public systems can distribute these interventions with fewer resources and to more parents.

The past decade has seen an increase in the number of randomized control trials (RCTs) examining the efficacy of parent-delivered early interventions for autistic children; however, most were conducted in specialized research clinics that often do not reflect how treatment is offered within community SLP programs [9]. This can negatively impact the generalizability of the findings to real-world clinical practice. While explanatory trials determine the efficacy of an intervention (in a highly controlled setting), *pragmatic* trials determine the effectiveness of an intervention in a real-world context, that is, everyday clinical practice. Pragmatic RCTs embrace the complexity, unpredictability, and inconsistency inherent in routine clinical practice by including diverse patient populations and allowing flexibility and variability in how the intervention is delivered [10]. Additionally, few clinical trials have included examination of for whom the intervention works (moderators of treatment outcome), why the intervention works (mediators of treatment outcomes), or how implementation processes could have impacted treatment effects. This leaves clinicians with limited information to effectively individualize and refine interventions to fit child, family, and system needs.

One parent coaching intervention commonly used by SLPs is *More Than Words®—The Hanen Program® for Parents of Autistic Children or Children Who May Benefit from Social Communication Support (MTW)* [11]. In fact, a recent survey [1] identified MTW as the preschool autism intervention program most widely used (61%) by SLPs in Ontario, Canada. The two existing RCTs on MTW involved university-based program delivery and report mixed findings that included positive effects in parents’ responsiveness and aspects of social communication for some children [12, 13]. Non-randomized observational studies of the MTW program reported improved parent responsiveness, self-efficacy, and interaction style; and improved child language and social communication skills post-program [14–16]. When asked about perceived outcomes, parents from community-delivered MTW programs highlighted the new strategies they learned, their child’s growth in functional communication skills, overall improvement in their relationship with their child, and the social and professional support network they gained [17]. Studies of parents’ experiences participating in MTW suggest that they value the program and the opportunity to connect with other parents, but seek improvements in navigating program content, the numerous forms associated with participation, parent-to-parent discussion, and the amount of individualized time with the SLPs [18, 19]. Although these results are promising, as is the case for parent-delivered interventions more broadly, community-based RCTs of the MTW program are lacking, and little evidence exists for the mediators and moderators of effective MTW delivery in real-world practice. Pragmatic RCTs are needed to build this understanding.

For a pragmatic RCT of the MTW program (and other parent coaching interventions) to be useful, it is critical to ensure that the methods used to evaluate treatment effects and mediators/moderators are not only sound but also ecologically valid and feasible for use in the community practice contexts in which the study is to be conducted. Given that real-world practice can be highly variable and unpredictable in ways that are outside of the control of the researcher, large-scale RCTs can be at risk for implementation failure without careful attention at the methodological planning stages. One solution is to first conduct a pragmatic methodological feasibility trial to identify and develop mitigation plans for possible issues in recruitment, retention, data collection, and analysis. A second associated solution is to employ principles of integrated knowledge translation, that is, ensure that co-construction and collaboration between researchers and community partners begins right from the planning stages. This can ensure that outcome measures selected are ideal from both a reliability, validity, and sensitivity perspective and practical for clinicians and families to use during real-world program delivery. In sum, for a full-scale pragmatic RCT to be successful and for results to be meaningful to end users, foundational work is needed that focuses on (a) systematic co-development of a comprehensive evaluation protocol and (b) pilot testing of the acceptability and feasibility of the evaluation protocol within real-world community settings.

With an overarching objective to conduct pragmatic RCTs of the MTW program, we first needed a comprehensive evaluation protocol of the MTW program as implemented in community practice that would be both feasible for clinicians and families and sensitive enough to measure treatment effects and moderators and mediators of child and family outcomes. Therefore, we partnered with program developers and expert SLPs from The Hanen Centre to co-construct a logic (causal) model [20] of the MTW program that links specific program objectives with evaluation tools (outputs, outcomes) and procedures [21]. In brief, key areas we integrated during development of the logic model and evaluation plan included the following: (a) theories and research guiding MTW program development; (b) outcome measures and treatment mediators and moderators identified in previous research; (c) expert SLP views on MTW objectives and outcomes, and mediators and moderators affecting treatment responses; and (f) organizational structures, barriers, and facilitators for SLPs delivering the program. The result of this co-construction process was a logic model of the MTW program and an associated evaluation protocol with proposed outcome measures.

The current paper addresses our second aim, which was to assess the feasibility of selected outcome measures and proposed evaluation procedures. Aligned with the framework proposed by Gadke and colleagues [22], we assessed the following dimensions of feasibility: (a) recruitment capability; (b) feasibility, acceptability, and practicality of data collection and analysis procedures; and (c) preliminary outcome trends based on the selected outcome measures.

## Methods

A pragmatic, single-arm, pre–post feasibility study was employed to evaluate the co-constructed evaluation protocol (see Table 1 for a summary). In alignment with our pragmatic intent, data were collected during real-world service delivery, that is, MTW programs delivered by community-based SLPs to families as a part of standard care.

**Table 1:** Summary of study objectives, outcomes, measures/approaches, and methods of analysis

Objective	Outcomes	Measures/approaches	Methods of analysis
1) Recruitment capability	Recruitment	– Number of eligible participants enrolled	– Descriptive statistics
2) Feasibility, acceptability, and practicality	Data collection and analysis procedures	– Completion rates for the study measures	– Descriptive statistics
		– Post-study semi-structured interviews of speech-language pathologists; ease of data collection	– Content analysis
		– Time required to complete video coding; interrater reliability	– Descriptive statistics; Cohen’s weighted Kappa
3a) Preliminary outcome trends: Child	Prelinguistic and early communication skills	– PPC (demographic data that can be used for sample description and moderator analysis in future RCT)	– Descriptive statistics
		– CFCS (communicative functioning that can be used for moderator analysis in future RCT); number and proportion of children in each category	– Descriptive statistics
		– CSBS-DP CQ; Total raw score, Social, Expressive Speech, and Symbolic Communication composite raw scores	– Means (SD), exploratory effect size estimates, 95% CI for effect size
	Communicative participation	– FOCUS-34; Total raw score; number and proportion of children in each clinical change category	– Means (SD), exploratory effect size estimates, 95% CI for effect size; Descriptive statistics
	Social communication stage & skills	– MTW SCC; number of children in each stage, and frequency of stage difference/change post program	– Descriptive statistics
	Overall social communication challenges and improvement	– CGI-S/CGI-I video coding; frequency and proportion of children in each category (rated on 7-point scales)	– Descriptive statistics
	Child interaction behaviours	– JERI video coding: rating on 7-point scale	– Means (SD), exploratory effect size estimates, 95% CI for effect size
	Perceived benefit for child	– Parent satisfaction survey; frequency and percentage (rated on 7-point scales)	– Descriptive statistics
3b) Preliminary outcome trends: Parent	Parent empowerment and strategy use	– Self-Efficacy/Knowledge Questionnaire; frequency and percentage (rated on 4-point scales); content analysis	– Descriptive statistics and content analysis
	Parent interaction behaviours	– JERI video coding: rating on 7-point scale	– Means (SD), exploratory effect size estimates, 95% CI for effect size
	Perceived helpfulness of program	– Parent satisfaction survey; frequency and proportion of parent responses (rated on 4-point scales); responses to open ended questions	– Descriptive statistics and content analysis
3c) Preliminary outcome trends: Dyad	Dyadic interaction	– JERI video coding: rating on 7-point scale	– Means (SD), exploratory effect size e, 95% CI for effect size

*PPC* Profile of Preschool Communication, *CFCS* Communication Function Classification System, *CSBS-DP CQ* Communication and Symbolic Behavior Scales – Developmental Profile, Caregiver Questionnaire, *FOCUS-34* Focus on the Outcomes of Communication Under Six, *MTW SCC* More Than Words Social Communication Checklist, *CGI-S/I* Clinical Global Impressions—Severity/Improvement, *JERI* Joint Engagement Rating Inventory

### MTW virtual program

MTW is a 13-week parent-implemented program designed to enhance communication, play development, and parent–child interaction for children under 4 years who have autism or social communication challenges. Traditionally delivered in-person, by MTW certified SLPs, a virtual version was added in 2020 [23] and was the model studied here. The virtual program includes an orientation session, a pre-program consultation appointment, eight 2½ h group training sessions (with 6–8 parents per group), and three individual video feedback sessions, all conducted via videoconference [11].

### Participants

In Ontario, Canada, parents with concerns about their young child’s speech-language development can self-refer for services through the publicly funded, provincial Preschool Speech and Language (PSL) program. For children with diagnosed or suspected autism in this program, the most used standard of care includes offering the MTW program to families [1]. All parent–child dyads in this study received MTW programs through the PSL program.

#### SLPs

The Hanen Centre invited MTW-certified SLPs from the PSL program known to be routinely offering MTW to families on their caseloads to participate. Interested SLPs were connected with the study team to assess eligibility, which required that SLPs (a) had previously delivered at least three MTW programs prior to the study, (b) were planning to offer a 13-week program in English between January 2022 and April 2023, and (c) were willing and able to support the planned data collection activities. A research team SLP attended the parent orientation session and introduced the opportunity to participate in the current study to all parents enrolled in the MTW program. Interested parents completed the informed consent process with a research team member over Zoom.

#### Parent–child dyads

As per standard care, parents were offered a MTW group program by their SLP based on the following eligibility criteria: the child (a) is diagnosed with autism, suspected of having autism, or identified as having social communication challenges; (b) is ≤ 47 months of age; (c) demonstrates delays in social communication and plays skills according to the SLP’s clinical assessment; and the parent (d) is available to attend the program and (e) is comfortable communicating in the language in which the program is being delivered. Where more than one parent of the same child was participating in the MTW program, one parent for each child was recruited to complete study measures. As per recommendations for non-randomized feasibility studies, a formal sample size calculation was not required [24] but we aimed to collect complete datasets from 12 parent–child dyads based both on Julious’ [25] recommendation of 12 participants per group for pilot feasibility trials, and what we assessed as practically possible within the study timeframe.

### Measures

#### Recruitment capability

We tracked the rates of study enrolment by SLPs and parents, and recorded reasons for SLP non-enrolment when these were offered. During the post-study interviews with enrolled SLPs, we gathered any known reasons for parents declining participation.

#### Feasibility, acceptability, and practicality of measures and procedures

We tracked completion rates for each study measure, that is, the number of dyads for whom data were provided to the research team. We also measured the time required for the research team to code videos and ability to attain sufficient coding reliability. Upon program completion, participating SLPs shared their perceptions on the barriers and enablers to recruitment and data collection, the practicality and useability of the outcome measures and procedures, and suggested design changes for a future RCT.

#### Preliminary outcome trends

As outlined in Table 1, our pragmatic evaluation protocol included measures of (a) baseline demographic and possible mediator/moderator variables; (b) a range of pre–post child, parent, and dyadic outcome variables; and (c) perceived effectiveness according to parents. Several of these measures are already administered by the SLP as standard care in the Ontario PSL or MTW programs. Although the inclusion of additional blinded measures would have increased the validity of the study’s results, considerations of parent burden, the research team’s capacity, and funding limitations precluded their inclusion. Measures are briefly described below (see clinicaltrials.gov/study/NCT06847347 for further details).

Baseline demographic variables were collected by the SLP pre-program using standard care measures: the *Profile of Preschool Communication (PPC) *and the* Communication Function Classification System* (CFCS). The PPC is an outcome monitoring tool designed within the World Health Organization’s International Classification of Functioning, Disability, and Health framework (ICF) [26] to collect information about preschool children’s communication and clinically relevant predictors of outcome and response to intervention [27–29]. The CFCS classifies a child’s everyday communication abilities into one of five levels based on how they send and receive messages [30, 31].

Child outcome measures administered pre- and post-program included the* Communication and Symbolic Behavior Scales-Developmental Profile Caregiver Questionnaire (CSBS-DP CQ), Focus on the Outcomes of Communication Under Six* (FOCUS-34), and *MTW Social Communication Checklist* (SCC). The CSBS-DP CQ contains 41 multiple-choice items rated by parents about a range of prelinguistic skills [32]. A total score of early communication skills is generated, along with three composite scores (social, speech, symbolic communication). The FOCUS-34 measures real-world changes in children’s communicative participation in response to speech-language intervention [33–36]. It includes 23 items on the child’s capacity for communication and 11 items on the child’s ability to engage independently, both rated by parents on a 7-point Likert scale. The total score ranges from 34 to 238 points, but the primary clinical outcome is the pre–post-program change scores. A change score of 11 points or more is considered a meaningful clinical change, 7–10 is possibly a meaningful clinical change, and 6 or less is not likely a meaningful clinical change [35]. The SCC is a Hanen Centre-developed checklist completed by SLPs in collaboration with parents to identify which of four stages best describe the child’s communication skills, coined by The Hanen Centre as *Own Agenda, Requester, Early Communicator, *and* Partner* [11]. We evaluated whether children stayed at the same stage or changed stages post-program. For children who stayed in the same stage, we also explored whether they gained new skills within that stage according to the SCC.

Two additional child outcome measures were coded from parent–child interaction videos recorded pre–post program. *Clinical Global Impressions* (CGI) coding pre-program assessed how impacted the child appeared by social communication challenges on a 7-point *Severity* (CGI-S) scale; coding post-program assessed changes in social communication challenges relative to pre-program on a 7-point *Improvement* (CGI-I) scale [37]. The *Joint Engagement Rating Inventory* (JERI) [38, 39] measured social engagement behaviors aligned with the MTW program. This included five child outcomes as well as four parent outcomes and two dyadic interaction outcomes. Each item is scored on a 7-point rating scale (from minimally to highly present).

Additional parent outcomes were collected pre–post program using the *Parent Self-Efficacy/Knowledge Questionnaire*. Two self-efficacy questions [40] asked parents to rate their confidence in helping their child to communicate and to develop their play skills, respectively, on a 7-point Likert scale (from strongly agree to strongly disagree). The two open-ended knowledge questions asked parents to list strategies or techniques they use to support their child’s communication and play skills, respectively.

Perceived effectiveness measures were collected from parents via the *Parent Satisfaction Survey* post-program. Parents rated statements and answered questions about whether, and to what extent, their child benefitted from the MTW program, they found the program to be helpful, and they changed how they interact and communicate with their child.

### Data collection procedures

SLPs administered, collected, and/or completed the PPC, CFCS, CSBS-DP, FOCUS-34, and SCC as per the typical procedures they follow during virtual service delivery (e.g., via email, over Zoom). They then uploaded anonymized copies of the measures via REDCap, a secure research data management platform housed at the university. The SLPs collected the videotaped parent–child interactions as per standard of care, then uploaded recordings to a secure university OneDrive folder for later JERI and CGI coding. Surveys that did not require collection or scoring by the SLP were delivered to parents directly via REDCap using the survey feature.

Both CGI and JERI coding was completed by a SLP researcher and a SLP trainee. To establish reliability, 4 of the 28 videos were double-coded in tandem; once agreement was reached, an additional 6 videos were independently rated by the two coders and reliability was calculated using weighted Cohen’s kappa [41]. Note that JERI coding occurred prior to CGI coding to mask video order (pre- vs post-program).

A 40-min virtual exit interview was conducted with each of the participating SLPs to explore their perceptions and gather suggestions. Interviews were conducted and recorded using Zoom for Healthcare by a trained researcher using a semi-structured interview guide. Field notes were used to extract information about feasibility and acceptability.

### Data analysis

Descriptive statistics were used to characterize participants at study onset and to summarize recruitment, retention, and data completion. Means and standard deviations were used for continuous variables (e.g., FOCUS-34 and CSBS-DP raw scores), and frequencies and percentages were used for categorical and scale variables (e.g., satisfaction, JERI codes, CFCS levels). This study was not intended or powered for detecting statistical effects; we report estimated effect sizes (using Cohen’s d) and their confidence intervals to support assessment of outcome trends and sensitivity to change of pre–post outcome measures. Descriptive and statistical analyses were completed using JASP version 0.19.1.0 [42].

Content analysis was used for SLP interview data and open-ended parent survey responses that related to the acceptability and feasibility of the study procedures and use of strategies. This method is well suited for use with small sample sizes as it can be used to quantify and describe participants’ experiences (e.g., attitudinal and behavioral responses to questions) [43].

## Results

### Participant demographics

Four SLPs participated, three who provide services in the Greater Toronto region and one in Western Ontario. All SLPs delivered the virtual version of the MTW program in English. Twenty-one parent–child dyads agreed to participate. Of the 16 parents who provided responses, 13 self-identified as the child’s mother and 3 as father.

Demographic information is provided in Table 2 for the 16 children for whom PPC forms and CFCS ratings were available. Due to an unintended error, child age was missing from most PPC forms. The ages of the five children for whom this was reported ranged from 22 to 35 months (*M* = 28.6, *SD* = 5.5) and the remaining were known to be at or below 47 months based on program eligibility criteria. Most children were male and all 16 had either a confirmed or suspected diagnosis of autism spectrum disorder; one child with suspected autism was also suspected of having global developmental delay. Half of the 16 children had a history of communication difficulties in their immediate family and roughly half attended a licensed daycare or school. No child was reported to have a home or environmental risk factors, and the majority were reported to have no personal factors associated with communication challenges. On the CFCS, most children were rated as level IV (inconsistent sender and/or receiver with familiar partners) or level V (seldom effective sender and receiver with familiar partners). One child each was rated as level III (effective sender and effective receiver with familiar partners) and level II (effective, but slower paced sender and/or receiver with unfamiliar and familiar partners).

**Table 2:** Demographics of enrolled children for whom PPC and CSBS forms were available

Variable	Total (%) *n* = 16
PPC	
Sex	
Male	12 (75)
Female	2 (12)
Not specified	2 (12)
Confirmed Biomedical Conditions	
Autism Spectrum Disorder	9 (56)
Suspected Biomedical Conditions	
Suspected Autism Spectrum Disorder	7 (44)
Suspected Global Developmental Delay	1 (6)
Immediate Family History of Communication Difficulties	
Language/Learning/Literacy	5 (31)
Speech	3 (19)
Weekly participation in an early learning environment	
Yes	9 (56)
No	7 (44)
Home environment and environmental risk factors	
None	16 (100)
Child factors associated with communication outcomes	
Behaviour difficulty or disorder	1 (6)
English/French not the child’s first language	2 (12)
Low birth weight	1 (6)
Other	2 (12)
None	10 (62)
CFCS	
Level II	1 (6)
Level III	1 (6)
Level IV	11 (69)
Level V	3 (19)

*PPC* Profile of Preschool Communication, *CFCS* Communication Function Classification System

### Feasibility outcomes

#### Recruitment capability

**Fig. 1:**
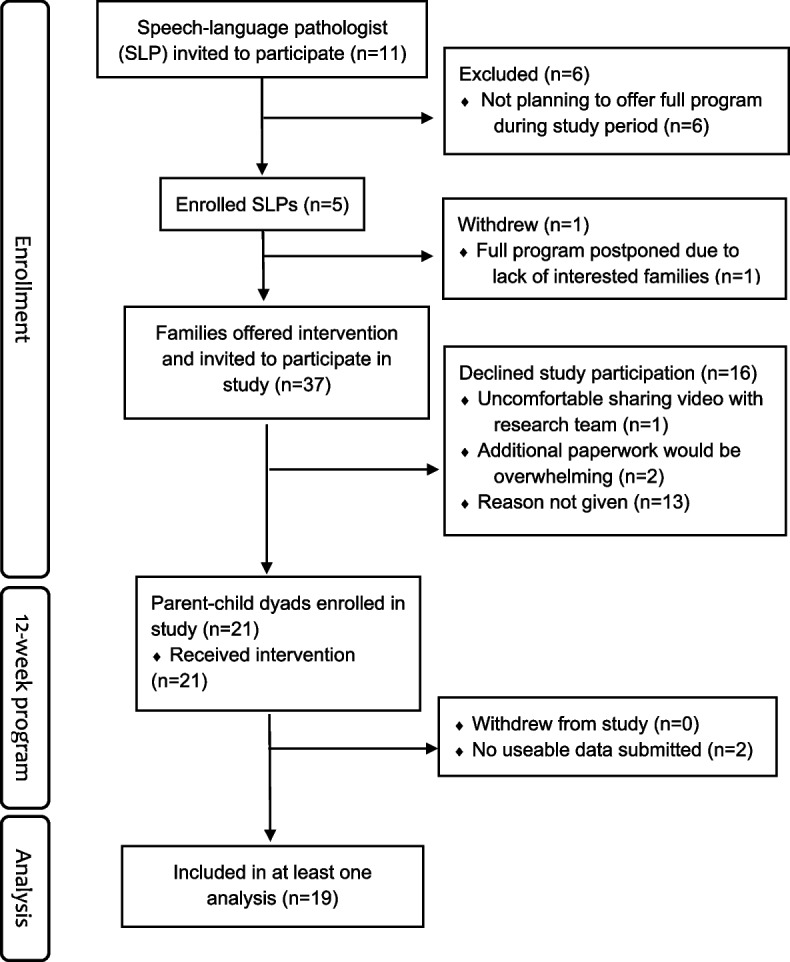
CONSORT flow diagram

Five SLPs from the Ontario PSL program of the 11 approached (45%) agreed to participate (see CONSORT flow diagram, Fig. 1). One SLP was unable to run her planned MTW program session during the study period due to insufficient enrolment following the orientation session. Thus, four SLPs (36% of those initially approached) ultimately participated and supported recruitment of parent–child participants. SLPs who did not agree to participate cited time constraints as the reason for non-participation. Specifically, they routinely delivered condensed MTW programs (e.g., 6–8 vs 13 weeks) and did not deem it clinically feasible to deliver the full 13-week program in the context of their caseload. All 37 parent–child dyads enrolled across the five MTW program sessions were approached by their SLPs about the opportunity to participate in the current study. Of these, 21 dyads (57%) agreed to participate. Reasons for non-recruitment revealed by the SLPs included one family indicating that they felt uneasy about potential privacy issues with sharing videos of their children and themselves. Two SLPs reported that families had expressed feeling overwhelmed with the prospect of the additional paperwork involved in the study. However, the SLPs reported that most families who elected not to participate in the study did not share their reasons for not participating.

#### Feasibility, acceptability, and practicality of measures and procedures

##### Measure completion rates

**Fig. 2:**
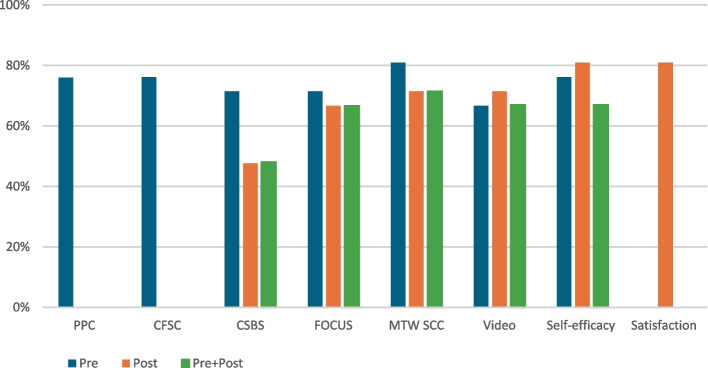
Proportion of dyads for whom data were successfully collected

Outcome measure completion varied according to the specific measure, the format of data collection for that measure, and timing (see Fig. 2 for a summary and Appendix for individual patterns). In general, 4 of the 21 dyads provided little to no data; as such, data completion peaked at 81% (17) for any one measure. Measures intended for pre–post analysis were generally collected at lower rates post-program relative to pre-program, with the exception of the Parent Self-Efficacy/Knowledge Questionnaire. To use pre–post measures for their intended purpose, data must be available for both time periods for the same dyad. In most cases, 60–70% of pre–post datasets were successfully collected with a notable exception for the CSBS-DP, which was below 50% due to lower post-program form submission compared to other measures. Of note, the forms completed online via REDCap by parents were among those with the highest submission rates (67–81%), suggesting that this mode of data collection was not a barrier for families. SLPs appeared equally successful in uploading data via REDCap versus OneDrive as indicated by similar patterns of data availability for these measures.

##### Coding

A second aspect of feasibility involved acceptability and practicality of coding the pre–post parent–child interaction videos with the CGI and JERI. In total, 6.05 h of video was watched by coders to complete the JERI and the same videos were viewed to complete the CGI. Video length averaged 23 min/participant for pre-program videos and 20 min for post. Following video review, it took coders an average of 5 min to code the CGI-S from a pre-program video and 3 min to code the CGI-I from the post-program video. Coding the 11 variables for the JERI took on average 15 min per video. The coders attained 0.67 reliability for the CGI-S and 0.72 on the CGI-I, indicative of a substantial level of agreement [41]. Inter-rater reliability for the individual JERI variables ranged from substantial to almost perfect agreement, with Cohen’s weighted Kappa values for the five child behavior items ranging from 0.71 to 1.0, the four caregiver items 0.70 to 0.90, and the two dyadic items 0.89 to 1.0.

The following factors were recorded as supporting the coding feasibility and reliability. First, coders were trained (or training) as SLPs. One was a clinician and researcher who has worked extensively in the field of autism for over 15 years, and the other was a student clinician who had experience working with preschool-aged autistic children and autism-focused interprofessional academic training. Second, coders took notes regarding the frequency, function, and means of the child’s communication. Doing so was reported to aid in establishing reliability and consistency in coding and enhanced the efficiency of conversations between coders when discrepancies in scoring arose.

##### SLP perceptions

Content analysis of the SLP exit interviews revealed that SLPs perceived the data collection process to be feasible. All mentioned that data collection procedures did not change much for them relative to what they typically do during MTW program delivery, apart from uploading of forms to the secure platforms (e.g., about 15 min/dyad total). All SLPs felt that the study measures were clinically meaningful. One SLP observed that families who participated in the study had higher and timelier form completion relative those who did not, which she reported was helpful as she often needs to inquire multiple times for forms to be completed. One SLP stated that uploading files via REDCap was cumbersome at first. She suggested that the usability of the data upload platform could be improved by withholding all file uploads until the end of the program and uploading them all at one time (post-program). To enhance future RCTs, one SLP suggested that researchers provide training for participating SLPs regarding how to videorecord the sessions, save videos in a single file, and upload them to a secure platform.

#### Preliminary outcome trends

Preliminary outcome trends were measured in two ways. First, patterns of change were compared across all pre–post outcome measures. Second, perceived effectiveness as reported by parents’ post-program was summarized.

##### Child outcomes

Children’s scores increased pre–post program in their overall prelinguistic and early communication skills (CSBS-DP total; see Table 3) as well as on the social, expressive speech, and symbolic composites on this measure. Most estimated effects were medium-sized but the 95% CI around these estimates included zero. A similar pattern was observed for children’s total score on the communicative participation measure (FOCUS-34). Analysis of FOCUS-34 change scores revealed that seven children (50%) showed a meaningful clinical change (∆FOCUS-34 ≥ 11), two (14%) a possible meaningful change (∆FOCUS = 7–11), and five (36%) likely no meaningful clinical change (∆FOCUS ≤ 6).

**Table 3:** Pre-post program changes in prelinguistic communication, communicative participation, and engagement

		Pre		Post			
	*n*	*M*	*SD*	*M*	*SD*	*d*	95% CI for *d*
Child Prelinguistic Communication (Communication and Symbolic Behavior Scales)							
Total score	10	71.6	31.1	82.5	31.1	.36	[-.29, 1.00]
Social composite	10	30.1	9.9	34.4	7.8	.46	[-.20, 1.10]
Expressive speech composite	10	16.4	7.6	18.9	7.5	.33	[-.31,.96]
Symbolic composite	10	25.1	14.8	29.2	12.7	.28	[-.36,.91]
Child Communicative Participation (Focus on the Outcomes of Communication Under Six)							
Total score	14	71.5	43.5	89.6	55.8	.33	[-.21,.86]
Child Engagement (Joint Engagement Rating Inventory; JERI)							
Initiation	14	2.6	0.8	3.5	1.0	.99	[.33, 1.62]
Responsiveness	14	2.0	0.9	4.1	1.0	2.20	[1.20, 3.18]
Expressive language	14	1.7	1.5	2.1	1.6	.23	[-.31,.76]
Behaviour patterns	14	1.9	1.1	3.4	1.0	1.48	[.70, 2.24]
Child affect	14	2.3	1.7	3.8	1.6	.89	[.26, 1.51]
Parent Engagement (JERI)							
Scaffolding	14	2.2	0.8	5.4	0.8	3.82	[2.27, 5.36]
Symbol highlighting	14	3.4	1.1	5.2	1.0	1.74	[.88, 2.57]
Following in	14	2.8	1.2	5.0	1.2	1.82	[.94, 2.68]
Parent affect	14	4.3	0.6	5.8	1.0	1.76	[.90, 2.60]
Dyadic Interaction (JERI)							
Fluency & connectedness	14	2.0	1.0	4.1	0.8	2.25	[1.23, 3.24]
Routines & rituals	14	2.4	1.1	4.8	0.8	2.43	[1.36, 3.48]

JERI ratings increased post-program for all five child activity items (see Table 3). All but the child’s expressive language showed large effects, with lower bounds of the 95% CI ranging from small to large effects. This included children’s initiation of communication, responsiveness to parent’s communication, quality of behavior patterns (i.e., moving from restricted or repetitive to varied and fluid), and affect (i.e., moving from disruptive or flat to mellow and content).

Most of the 15 children with SCC pre–post data were assessed as being at the Requester stage pre-program in terms of both the reasons why they communicate (73%) and the ways in which they communicate (67%; see Table 4). Most children stayed at the same stage but gained additional skills within that stage, most often in the ways in which they communicate. A small number of children (13–20%) moved up a stage. No child lost skills or moved down a stage.

**Table 4:** Changes in children’s social communication stages and skills pre-post program

	Social communication stage difference			
Pre-program stage	Same (=)	Same (+)	Moved up	*n*
Reasons Child Communicates				
Own Agenda	0	0	0	0
Requester	9	0	2	11
Early Communicator	1	1	1	4
Partner	0	1	0	1
Total	10	2	3	15
Ways Child Communicates				
Own Agenda	0	0	1	1
Requester	2	8	0	10
Early Communicator	1	1	1	3
Partner	0	1	0	1
Total	3	10	2	15

Stage Difference corresponds to stages and skills on the Social Communication Checklist; Same (=) = stayed at the same stage and did not gain any new skills; Same (+) = stayed at the same stage but gained new skills within that stage; Moved up = went up one stage

All 17 parents who completed the satisfaction survey indicated that they strongly agreed (*n* = 12, 71%) or agreed (*n* = 5, 29%) that their child benefitted from the MTW program. In alignment with parent perceptions, all researcher-coded CGI-I ratings based on available videos (*n* = 14) indicated a global impression of social communication improvement. Pre-program CGI-S ratings indicated that 21% of children were severely impacted in their social communication challenges, 43% were markedly impacted, 21% moderately, and 14% mildly. Post-program, 14% were rated on the CGI-I as very much improved, 36% as much improved, and 50% as minimally improved, with no child’s social communication challenges being rated as showing no improvement or worsening relative to pre-program severity ratings.

##### Parent outcomes

Parents demonstrated higher mean JERI ratings post-program on all four items related to their interaction with their child (see Table 3). Effects’ size estimates were large across the 95% CI. Parents increased their scaffolding (i.e., provision of supports of their child’s activities and opportunities for learning and communicating), highlighting (e.g., attempts to direct their child’s attention to words or gestures), following in on their child’s focus, and affect (i.e., moving from tense or expressionless to more mellow and content).

On average, parents who completed both pre–post-program self-efficacy questionnaires (*n* = 14) reported increased confidence post-program in their ability to help their child to communicate, and to develop their play skills (see Table 5). The proportion of parents agreeing or strongly agreeing that they feel confident in supporting their child’s communication was notable, increasing from 38% pre-program to 79% post-program. Although only three parents expressed limited self-confidence in supporting their child’s play skills pre-program, 100% agreed or strongly agreed that they felt confident to do so post-program.

**Table 5:** Changes in parent self-efficacy pre-post program

	I feel confident I can help my child communicate their thoughts and ideas *n* (%)	I feel confident I can help my child develop their play skills *n* (%)
Pre-program		
Strongly agree (1)	2 (14)	5 (38)
Agree (2)	3 (21)	6 (43)
Somewhat agree (3)	7 (50)	2 (14)
Neither agree nor disagree (4)	4 (29)	0
Mean rating (*SD*)	2.5 (1.0)	1.7 (0.7)
Post-program		
Strongly agree (1)	3 (21)	9 (64)
Agree (2)	8 (57)	5 (38)
Somewhat agree (3)	2 (14)	0
Neither agree nor disagree (4)	1 (7)	0
Mean rating (*SD*)	2.0 (0.8)	1.4 (0.5)

Content analysis of parents’ survey responses about strategies and techniques they use to support their child’s communication pre-program focused on responsive interaction and structured techniques. Common strategies included modeling, repetition, engagement through play, visual/tactile supports, nonverbal cues, emotional attunement, and structured interactions. Pre-program responses on strategies to support their child’s play skills included child-led play, imitation, modeling, turn-taking, sensory play, and structured and repetitive play. Responses to the same questions post-program heavily referenced specific strategies from the MTW program, including R.O.C.K. (repeat, offer opportunities, cue, and keep it going), the Four I’s (include the child’s interest, interpret, imitate, and introduce more fun), and O.W.L (observe, wait, listen). In their post-program communication strategies, parents also emphasized simplified and clear communication, face-to-face interactions, incorporating their child’s interests, modeling, and open-ended opportunities. In their post-program play strategies, parents also emphasized observing and responding to their child’s cues and engaging at their level, including gently expanding on their solitary play. Overall, while there was overlap in some pre- and post-program strategies (e.g., modeling, child-led play), post-program strategy use reported by parents focused more on specific MTW program strategies and interactive, responsive, child-centered supports, which highlighted fidelity to treatment and new knowledge gained through the MTW program.

Of the 17 parents reporting perceived effectiveness of the MTW program for themselves, all but one found the program helpful (94%). Strategies they reported as most helpful included “Follow your child’s lead using the Four I’s” (94%) and “Identifying your child’s communication stage” (88%). Play strategies like “R.O.C.K. in people games and songs” and “R.O.C.K. with people toys” were also frequently well received (77%). Fewer parents found “Helping your child make friends” helpful (29%).

##### Parent–child interaction outcomes

The parent–child dyads demonstrated higher JERI ratings of their joint engagement during the video interactions post-program, with large effects observed across the 95% CI for both dyadic interaction outcome measures (see Table 3). That is, parent–child interactions and/or communicative exchanges increased, and routines and rituals became more frequent and varied.

All 17 parents who completed the Satisfaction Questionnaire attributed changes in the way they interact and communicate with their child to their participation in the MTW program. When asked to list the most important changes, several key themes emerged (see Table 6). Under the theme of enhanced engagement, parents reported becoming more animated, vocal, and silly, making interactions more enjoyable. Parents emphasized maintaining eye contact, keeping interactions fun, and being consistent, which sustained their child’s interest. A few parents mentioned positively intruding on their child’s play. They noted paying more attention to their child’s needs and providing opportunities for imitation, exploration, and turn-taking. Within the theme of improved communication, parents noted having more face-to-face interactions, better interpretation of messages, and more imitation of their child’s actions. Specific communication strategies were highlighted by some parents such as using smaller phrases, leaving space for communication, and making comments that invited responses. Several parents learned to wait and provide cues for turn-taking to encourage their child to make more requests. Parents reported asking fewer questions and making more inviting comments to promote better communication. Some parents emphasized “getting down to their child’s level” to better understand their interests in order to interpret and respond more effectively. Lastly, parents reported feeling more relaxed and better equipped to identify effective strategies when their child faced communication or play difficulties, enabling a more supportive environment.

**Table 6:** Self-reported improvements in parent–child interaction and communication

Key themes	n (%)
Enhanced Engagement	
Consistency and Fun	8 (47)
Positive Play Intrusion	5 (29)
Attention to Child's Needs	4 (24)
Improved Communication	
Communication Strategies	7 (41)
Encouraging Requests and Turn-Taking	6 (35)
Reduced Questioning	6 (35)
Better Understanding of Child's Interests	4 (24)
Relaxation and Empowerment	4 (24)

## Discussion

The purpose of the present study was to test the methodological feasibility and acceptability of an evaluation protocol of the MTW program delivered within real-world community settings and engage in a preliminary exploration of outcome trends. Using outcome measures and procedures that were co-constructed with program developers and SLPs who had extensive expertise and experience in delivering the program [21] meant that the protocol was set up for success from both scientific and practicality perspectives. We assessed three dimensions of feasibility [22]: (a) recruitment capability; (b) feasibility, acceptability, and practicality of our data collection and analysis procedures; and (c) preliminary outcome trends.

### Recruitment capability

We were successful in recruiting community SLPs and eligible parent–child dyads. Roughly one-third of the SLPs approached were planning to deliver a 13-week MTW program session as a part of their routine clinical service delivery and agreed to participate, and just over half of enrolled families consented to the trial. Recruitment rates were similar to those in previously published pediatric pilot intervention studies with autistic children (e.g., [44, 45]). Although it is impossible to fully predict the enrollment rates for a future RCT, those we encountered here provide a useful estimation for planning purposes.

Although five SLPs initially agreed to support the recruitment of parent–child dyads, one (20%) SLP withdrew due to an insufficient number of families enrolled in the program during the study timeframe. This type of retention issue will be an additional consideration for future pragmatic trials, namely, that the need for an interconnected cluster of participants (i.e., parent, child, and their SLP) within studies of parent-mediated programs increases the complexity of recruitment. No families withdrew; however, completion rates of outcome measures were lower than expected (e.g., 60% for some measures). It is possible that completion rates reflected some families reducing engagement in the MTW program without this being directly reported to the research team. The participating SLPs indicated that form completion challenges are commonly experienced, suggesting that our study retention rates reflected real-world community engagement. Overall, we might anticipate a minimum level of 20% missing data in future pragmatic trials. Of note, within the context of a RCT, we would be in a position to compare pre-program profiles of families for whom we have versus lack post-program data to better understand contributing factors, which was not possible in this feasibility trial due to the low sample size.

### Feasibility, acceptability, and practicality of measures and procedures

This study provided valuable insights into the feasibility, acceptability, and practicality of our outcome measures and procedures. One of the greatest learnings related to our success rates and challenges in getting the data into the hands of the research team. No measure achieved more than 80% completion, with most pre–post form datasets 60–70% complete. As above, SLPs reported that our completion rates were consistent with what they experience in routine clinical practice and that study participation did not generally appear to negatively impact parent response rates. In fact, one SLP reported that it enhanced completion rates. Of interest, both the parent self-efficacy and knowledge questionnaires (completed via online direct survey links) were among the measures with the highest completion rates. By contrast, forms collected at the point of care (e.g., PPC, CSBS, CFCS, FOCUS-34) had lower completion rates. This raises the possibility that providing parent forms via an online platform (i.e., REDCap) accessible via mobile devices may serve as a facilitator for some families. Previous research has demonstrated that addressing logistical barriers, such as travel distances, can significantly improve retention rates in intervention studies with autistic children [46]. Although the current study involving virtual delivery eliminated the need for travel, a similar principle—enhancing accessibility via online surveys for data collection—can be effectively applied to virtual contexts and may further support data completion and retention rates.

It was unclear which SLP and/or parent factors specifically contributed to failure of form completion or data upload in each instance of missing data. Missing data may be expected in a pragmatic trial in which the research team is not involved in directly administering the measures to the participants. We were not resourced to closely monitor measure completion or data submission throughout the trial, or to provide participant reimbursement, consistent with our pragmatic aim to assess the feasibility of obtaining outcome without additional researcher-initiated follow-up. Consequently, the completion rates reported here may represent a conservative estimate of what is achievable. In a future RCT, it is reasonable to expect that adding routine data monitoring, researcher collection of pre–post measures, enhanced participant engagement strategies and supports, and incremental participant reimbursement, might support higher data completion rates. At minimum, the current findings provide a useful starting point for data collection expectations and possible mitigation strategies for a future pragmatic RCT.

Our findings also supported the feasibility of applying our two coding measures, the CGI and JERI, to pre–post parent–child interaction videos collected as a part of the MTW program. We found the time required to view and code the videos to be acceptable from a resourcing perspective, with coders requiring on average 45 min to view and complete both CGI and JERI coding for one video. Coding was reliable, with substantial to almost-perfect agreement between coders on both measures [41]. Finally, we learned valuable insights on coder skillset and strategies to enable practicality and feasibility of these coding procedures for a future RCT.

Finally, participating SLPs generally found the outcome measures to be clinically meaningful and the data collection procedures to be reasonable. They reported additional time requirements for data upload to be manageable and the upload procedures to be acceptable. This overall positive response was likely supported by our use of a co-design approach in development of the evaluation procedures and measures, which has been found to positively impact study quality and participant satisfaction [47].

### Preliminary outcome trends

Children for whom data were available showed positive increases from pre- to post-program in their overall prelinguistic and early communication skills, communicative participation, and engagement during interactions with their parent. All children either moved up in their social communication stage or gained skills within their current stage and showed some degree of improvement in the overall severity of their social communication challenges. All children were perceived to have benefited from their program by their parent and a majority showed a clinically meaningful or possibly meaningful change in their communicative participation post-program. These observations were made across measures collected by different means (parent report, SLP rating, researcher coding).

Parents in this trial for whom data were available also demonstrated positive increases across measures. Observational measures collected post-program showed improvements in all aspects of parents’ engagement activities during interactions with their child and in parent–child joint engagement. Furthermore, many parents reported increased self-efficacy around their ability to support their child’s communication and most perceived the MTW program as helpful. In alignment with findings from a qualitative study exploring parents perceived outcomes of the MTW program [17], parents in the current study reported their interaction and communication with their child changed as a result of the program. As such, both the observed and perceived outcome trends of the MTW program in the parent and dyadic domains targeted by the program were captured using the measures from our evaluation protocol.

The almost uniform pattern of positive raw score change found for our pre–post program measures supports the potential of the selected measures to be sensitive to change in real-world practice and thus be suitable for use in a future pragmatic RCT. These findings align with those of prior pre–post observational studies that also reported positive changes following the MTW program in children’s social communication, parent responsiveness, and parent self-efficacy [14, 15]. The estimate effect sizes and 95% CI around these provided some indication that our selected measures, in particular the JERI, may be reasonable for change detection in a RCT; however, it is critical to caution that these comparisons only measured change over time.

It remains to be seen whether the resolution of our outcome measures will be sufficient to differentiate changes *during* intervention from any changes *attributable* to intervention. This will only be discoverable through controlled designs like RCTs that include a comparison group that is not receiving the MTW program. Both prior RCTs of the MTW program also found positive gains pre–post program in their child and parent outcome variables, but the extent to which these were associated with treatment effects varied. Carter et al. [12] found positive raw score changes in children’s frequency of initiating joint attention, initiating behavior requests, and intentional communication, and in parents’ nonverbal responsivity; however, these gains only represented a treatment effect for children with low object interest pre-program and in only some aspects of their communication; other gains were observed in the control group as well. By contrast, in a pilot RCT of an abbreviated version of the MTW program, Venker et al. [13] found gains in children’s prompted communication and in parents’ responsive communication behaviors that did reflect a treatment effect relative to the control group. Variable findings might be expected given these two RCTs differed not only in the duration of their MTW programs but also in the mean age of the child participants, sample sizes powering their pre–post analyses, and the specific child and parent outcome measures used. On the latter point, the outcome measures examined here are the first to be generated from a co-constructed logic model of the MTW program and its intended objectives and outcomes; a future pragmatic RCT using this protocol may be used to rigorously evaluate outcomes of the MTW program in community settings.

### Future directions

Collectively, the results of this feasibility trial provided support for use of our co-constructed evaluation protocol in a future RCT of the MTW program. We believe that the most reasonable next step is to proceed to a pilot rather than full-scale RCT. In a pilot RCT, we will be able to benefit from a priori estimations of recruitment, retention, and adherence rates generated here and observe how new adjustments and procedures we will develop in response to these alter our success. A pilot RCT will also provide us with critical information on recruitment, implementation fidelity, retention, and data collection for the comparison group (e.g., waitlist control), which we did not assess here and for whom methodological uncertainties remain.

For convenience purposes, we conducted this feasibility trial within the context of the Ontario PSL program. Although the MTW program sessions delivered during this trial were associated with two different regions offered by four different SLPs, they were all provided as a part of the same publicly funded service and delivered virtually. This is not necessarily representative of the broader range of community settings in which the MTW program is delivered. Moreover, the Ontario PSL program included two measures (CFCS, FOCUS-34) collected as a part of routine care that we were able to leverage for this study. A pilot RCT would allow us to evaluate the applicability of our protocol to other jurisdictions and with SLPs who do not already use some of the outcome measures in their clinical practice.

## Conclusions

The present feasibility trial provides a novel contribution to the scientific and clinical communities by highlighting the methods, procedures, challenges, and benefits of embarking on pragmatic clinical trial work in the field of social communication interventions for young children and their families. We acknowledge that it can be difficult for researchers to step outside of the control afforded by experimental designs and controlled settings, but our view is that the benefits and promise for end users, and for our own science, far outweigh the challenges. While specific outcomes from this feasibility study may not directly transfer to other contexts, insights gained through exploring the methodological feasibility and acceptability of recruitment and data collection in this study extend beyond the MTW program, providing valuable methodological considerations for future pragmatic trials of other caregiver-mediated programs.

A critical gap remains in our understanding of the benefits of parent training programs designed to support autistic children and others with social communication challenges as they are implemented in routine clinical practice, as well as in our understanding of for whom such interventions work best, in which ways, and why. This impacts our ability to be fully evidence-informed in service delivery and in supporting choices for families. Carefully designed, ecologically valid, pragmatic evaluation protocols, feasibility trials, and pilot studies will help us begin to fill this gap together.

## Data Availability

The participants of this study did not give written consent for their data to be shared. Given this and the sensitive nature of the research, data are not available for sharing.

## Appendix

Form completion and video availability for each parent-child dyad

**Table:**

	1	2	3	4	5	6	7	8	9	10	11	12	13	14	15	16	17	18	19	20	21
PPC	✓	✓	✓	✓	✓	✓	✓	✓	✓	✓	✓	✓	✓	✓	✓	✓					
CFCS	✓	✓	✓	✓	✓	✓	✓	✓	✓	✓	✓	✓	✓	✓	✓		✓				
CSBS T1	✓	✓	✓	✓	✓	✓	✓	✓	✓	✓	✓	✓	✓	✓	✓						
CSBS T2	✓	✓	✓	✓			✓	✓	✓	✓	✓	✓									
FOCUS T1	✓	✓	✓	✓	✓	✓	✓	✓	✓	✓	✓	✓	✓	✓	✓						
FOCUS T2	✓	✓	✓	✓	✓	✓	✓	✓	✓		✓	✓	✓	✓	✓						
SCC T1	✓	✓	✓	✓	✓	✓	✓	✓	✓	✓	✓	✓	✓	✓	✓	✓	✓				
SCC T2	✓	✓	✓	✓	✓	✓		✓	✓	✓	✓	✓	✓	✓	✓	✓					
Video T1	✓	✓	✓	✓	✓	✓	✓	✓	✓	✓		✓	✓			✓	✓				
Video T2	✓	✓	✓	✓	✓	✓	✓	✓	✓	✓		✓	✓			✓	✓			✓	
Parent self-efficacy T1	✓	✓	✓	✓	✓	✓	✓			✓	✓	✓	✓	✓		✓	✓	✓	✓		
Parent self-efficacy T2	✓	✓	✓	✓	✓	✓	✓	✓	✓	✓	✓			✓	✓	✓	✓	✓	✓		
Parent satisfaction	✓	✓	✓	✓	✓	✓	✓	✓	✓	✓	✓			✓	✓	✓	✓	✓	✓		
Total	14	14	14	14	13	13	13	13	13	12	12	12	11	11	10	8	7	3	3	1	0

*PPC* Profile of Preschool Communication, *CSBS* Communication and Symbolic Behavior Scales, *T1 *Time 1, *T2* Time 2, *CFCS* Communication Function Classification System, *FOCUS* Focus on the Outcomes of Communication Under Six, *SCC* Social Communication Checklist

## Abbreviations

CFCS: Communication Function Classification System
CGI-S/I: Clinical Global Impressions—Severity/Improvement
CSBS-DP: Communication and Symbolic Behavior Scales – Developmental Profile
CQ: Caregiver Questionnaire
FOCUS: Focus on the Outcomes of Communication Under Six
ICF: International Classification of Functioning, Disability, and Health framework
JERI: Joint Engagement Rating Inventory
MTW: More Than Words®—The Hanen Program® for Parents of Autistic Children or Children Who May Benefit from Social Communication Support
PACT: Preschool Autism Communication Trial
PPC: Profile of Preschool Communication
PSL: Preschool Speech and Language
RCT: Randomized control trial
REB: Research Ethics Board
SCC: Social Communication Checklist
SLP: Speech-language pathologist
